# Comparative outcomes of combined thermal ablation and liver resection versus liver resection alone for multiple colorectal liver metastases: a systematic review and meta-analysis

**DOI:** 10.3389/fonc.2025.1613615

**Published:** 2025-06-27

**Authors:** Zesong Meng, Baokun Li, Chaoxi Zhou, Longfei Cao, Jianfeng Zhang, Jun Feng, Guiying Wang

**Affiliations:** ^1^ Department of General Surgery, Fourth Hospital of Hebei Medical University, Shijiazhuang, Hebei, China; ^2^ Department of General Surgery, The Second Hospital of Hebei Medical University, Shijiazhuang, China

**Keywords:** liver resection, meta-analysis, colorectal cancer, liver metastases, thermal ablation

## Abstract

**Background:**

The treatment of colorectal liver metastases (CRLM) continues to pose a significant clinical challenge, with surgical resection remaining the gold standard. However, the efficacy of combining thermal ablation (TA) with liver resection (LR) compared to LR alone in managing multifocal CRLM remains a topic of debate. This meta-analysis aims to compare the outcomes of combining TA and LR with LR alone in patients with multifocal CRLM.

**Methods:**

A comprehensive literature search was conducted across PubMed, EMBASE, Cochrane Library, and Web of Science up to December 2024. Studies that compared the combination of TA and LR with LR alone in patients with CRLM and reported at least 1-, 2-, or 3-year overall survival (OS) and/or disease-free survival (DFS) were included. Data were extracted and analyzed using random-effects or fixed-effects models, depending on the degree of heterogeneity. Sensitivity analysis and assessment of publication bias were performed to ensure the robustness of the findings.

**Results:**

Six retrospective cohort studies involving 3084 patients (1286 in the TA+LR group and 1798 in the LR group) were included. No significant differences were found in 1-, 2-, and 3-year OS between the TA+LR and LR groups. However, the TA+LR group exhibited worse DFS. Subgroup analysis revealed a more pronounced decline in DFS in non-European TA+LR cohorts compared to LR cohorts, potentially reflecting regional differences. Additionally, DFS was significantly lower in the radiofrequency ablation (RFA) subgroup compared to the microwave ablation (MWA) subgroup. Complication rates were comparable between the two groups. Sensitivity analysis confirmed the stability of the results, and no significant publication bias was detected.

**Conclusion:**

Combining thermal ablation with liver resection is a feasible liver-sparing approach for treating extensive CRLM, applicable through both laparoscopic and open surgical techniques. Combined resection and ablation should be considered as an alternative to resection alone for patients with multiple metastases.

**Systematic review registration:**

PROSPERO https://www.crd.york.ac.uk/prospero/, identifier CRD42024629343

## Introduction

Colorectal cancer (CRC) is a prevalent malignancy, ranking fourth globally in terms of incidence and third in cancer-related mortality, as reported by the World Health Organization (1, 2). Notably, approximately 22% of CRC cases are diagnosed with metastatic disease, which dramatically reduces life expectancy and causes a significant drop in 5-year survival rates from 90% for localized disease to 15% for those with distant metastases (3). While curative-intent hepatectomy is considered the optimal treatment for colorectal liver metastases (CRLM), only a limited number of newly diagnosed patients are candidates for surgical resection. This is often due to factors such as unresectable tumor burden, inadequate future liver remnant, or compromised functional status. In such cases, cytoreductive chemotherapy is frequently employed as a bridge to potentially resectable disease (4).

Achieving the best oncologic outcomes in CRLM often requires a multimodal approach, including radical hepatectomy. This may involve multistage hepatectomy (MSH), portal vein embolization (PVE), associating liver partition and portal vein ligation for staged hepatectomy (ALPPS), thermal ablation (TA), chemoembolization, or perioperative chemotherapy (5). Thermal ablation (TA) has gained prominence as both an adjunctive and standalone treatment option in CRLM management, with applications in both intraoperative and percutaneous settings (6, 7). Modern ablation techniques, such as radiofrequency ablation (RFA) and microwave ablation (MWA), have been increasingly utilized (8). These advancements have broadened the role of TA in multidisciplinary protocols, as evidenced by its inclusion in current CRLM treatment guidelines (9, 10).

Despite these developments, the evidence supporting the combination of resection and ablation for multifocal CRLM remains limited and heterogeneous, often compromised by methodological limitations (11, 12). Comparative analyses are frequently based on studies involving TA for unresectable lesions, which may introduce selection bias in survival outcomes. The scarcity of robust randomized controlled trials (RCTs) and prospective cohort studies further complicates the determination of optimal treatment strategies.

This study aims to evaluate the efficacy and safety of combining hepatectomy with TA versus hepatectomy alone in the management of multifocal CRLM. It seeks to provide insights for clinical decision-making and guide future research directions in the treatment of metastatic CRC.

## Methods

This meta-analysis was conducted in accordance with the PRISMA 2020 and AMSTAR guidelines to ensure methodological rigor. The protocol was registered on PROSPERO (CRD42024629343).

### Literature search

A comprehensive search was performed across major databases, including PubMed (Medline), EMBASE, Cochrane Library, and Web of Science, to identify relevant studies published up to December 2024. The search terms included “Microwave ablation” (MWA), “Thermal ablation” (TA), “Radiofrequency ablation” (RFA), “Liver resection” (synonyms: “Hepatic resection,” “Hepatectomy”), “Colorectal” (synonyms: “Colon,” “Rectal”), “Cancer” (synonyms: “Tumor,” “Carcinoma”), and “Liver metastasis” (synonyms: “Hepatic metastases”). Only English-language publications were considered. Additionally, the reference lists of retrieved articles were manually reviewed to identify additional relevant studies.

### Inclusion criteria

Studies were included if they met the following criteria: (1) they compared the clinical outcomes of combined thermal ablation (TA) with liver resection (LR) versus LR alone for treating colorectal cancer liver metastases (CRLM); (2) they reported at least 3- or 5-year overall survival (OS) and/or disease-free survival (DFS) for each treatment group; (3) in cases of multiple publications from the same research group, only the most recent and comprehensive study was included; (4) studies involved patients with CRLM (preoperatively or intraoperatively diagnosed with liver metastases); and (5) only randomized controlled trials (RCTs) and non-RCTs published in English were included.

### Exclusion criteria

Studies were excluded if they: (1) did not provide comparative data on the therapeutic efficacy of MWA versus HR; (2) were unsuitable publication types (case reports, conference abstracts, meta-analyses, reviews, or animal experiments); (3) included patients with extrahepatic metastases; (4) lacked a control group or had an unreasonable control group; (5) were not written in English; (6) were of low quality; or (7) no original data could be obtained from the corresponding author.

### Quality assessment

The methodological quality of the included studies was assessed using the Newcastle-Ottawa Scale (NOS). Two independent reviewers (Zesong Meng and Baokun Li) evaluated the studies, and any disagreements were resolved through consultation with a third reviewer (Longfei Cao). Studies scoring ≥6 on the NOS were deemed high quality.

### Data extraction

Data extraction was independently conducted by Baokun Li and Chaoxi Zhou, with discrepancies resolved through discussion with a third reviewer (Longfei Cao). Extracted data included: (1) study characteristics (first author, publication year, sample size, study location, and design); (2) baseline oncological characteristics; and (3) clinical outcomes (1-, 2-, 3-, and 5-year OS and DFS, as well as perioperative outcomes comparing resection and combined resection and ablation).

For the meta-analysis, when individual patient-level data were available, Kaplan-Meier curves and risk tables were reconstructed. For studies lacking primary data, summary data were extracted from published Kaplan-Meier curves, including treatment group details, overall survival probabilities with corresponding time points, and recurrence-free survival probabilities with associated time intervals (10, 11).

### Statistical analysis

Dichotomous variables were analyzed using odds ratios (OR) with 95% confidence intervals (CI). Inter-study heterogeneity was assessed using I² statistics. Time-to-event data, including 1-, 2-, and 3-year OS, were extracted from individual studies. Pooled categorical comparisons were conducted using the Chi-squared test. A random-effects model (DerSimonian-Laird method) was applied if significant heterogeneity (I² > 50%) was observed; otherwise, a fixed-effect model was used. A two-sided p-value < 0.05 was considered statistically significant. Sensitivity analysis was performed by sequentially excluding each study to assess the stability of the results. Publication bias was evaluated using Begg’s and Egger’s tests. All analyses were conducted using STATA version 12.0.

## Results

### Study characteristics and patient demographics

**Figure 1:**
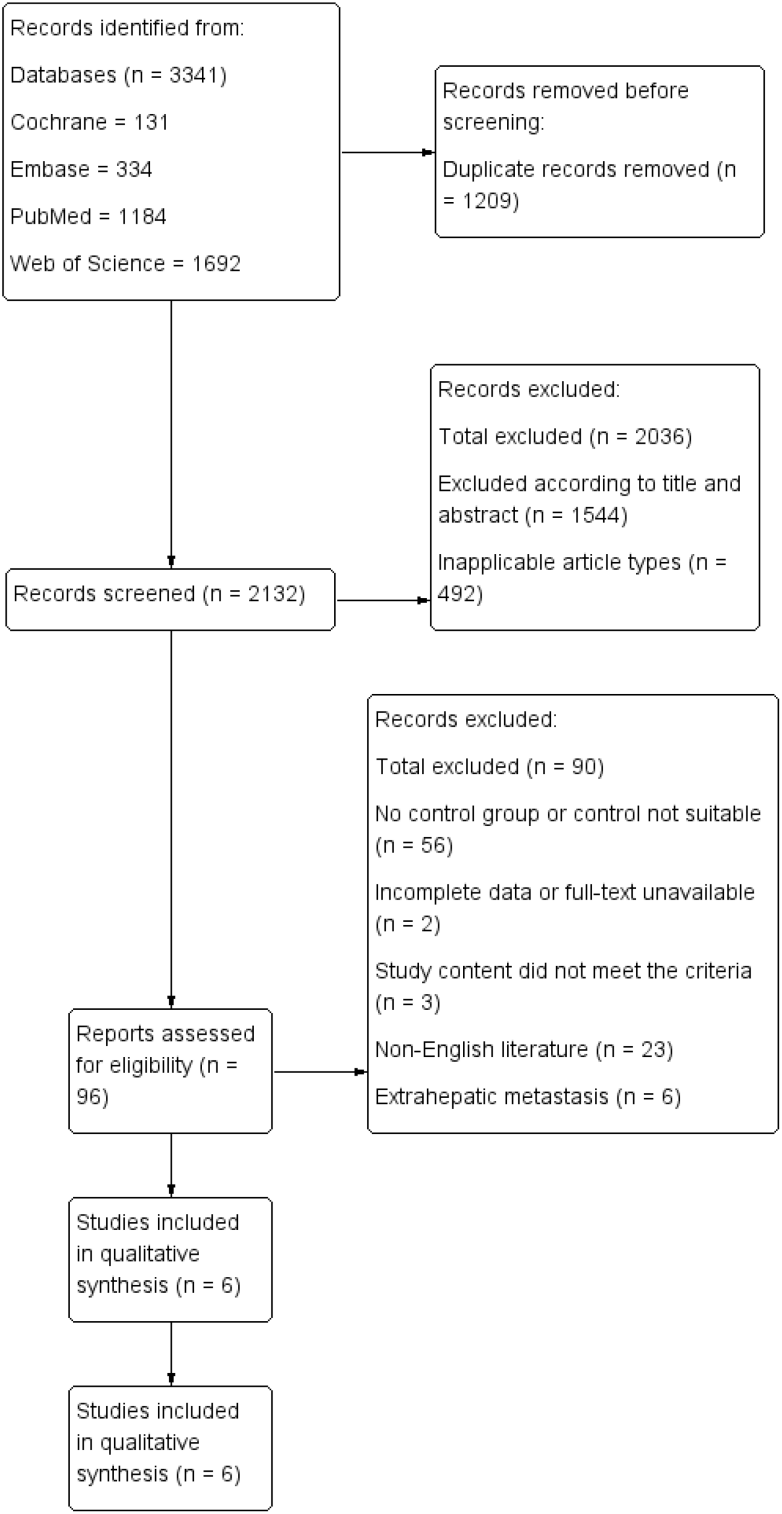
The flowchart describing the selection of the literature.

A total of six retrospective cohort studies, involving 3,084 patients (1,286 in the TA+LR group and 1,798 in the LR group), were included in this meta-analysis (**Figure 1**). The final analysis encompassed six retrospective cohort studies (11–16). All studies demonstrated high methodological quality, achieving Newcastle-Ottawa Scale (NOS) scores of at least 7. The baseline characteristics of the included studies are detailed in **Table 1**. The majority of these studies were conducted in Europe (n=4), while the remaining two studies originated from China and the United States.

**Table 1:** Characteristics of the included studies.

Study	Country	Type of study	Comparison	LR	TA+LR	Tumor number	Total sample
Eddie 2004	America	Retrospective study	RFA+LR *VS* LR	190	101	NA	291
Fabio 2023	France	Retrospective study	RFA+LR *VS* LR	71	57	NA	128
Michelle 2023	Netherlands	Retrospective study	TA+LR *VS* LR	1005	1005	≥ 2	2010
Yunzhu 2021	China	Retrospective study	MWA+LR *VS* LR	380	57	NA	437
Iakovos 2023	Germany	Retrospective study	TA+LR *VS* LR	132	46	≥ 4	178
Simone 2022	Italy	Retrospective study	MWA+LR *VS* LR	20	20	NA	40

### Overall survival

**Figure 2:**
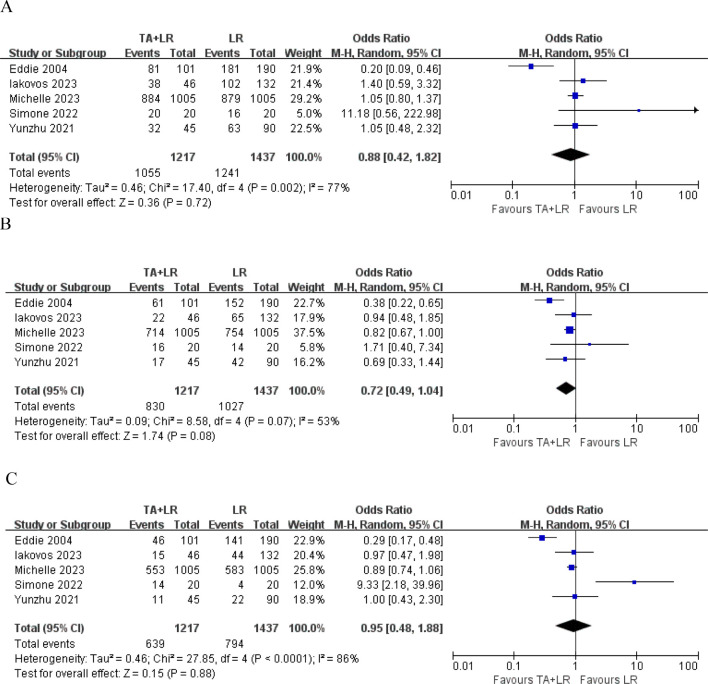
Forest plots comparing overall survival rates between TA+LR and LR groups. **(A)** Pooled analysis of the 1-year overall survival rate. **(B)** Pooled analysis of the 2-year overall survival rate. **(C)** Pooled analysis of the 3-year overall survival rate. OR, Odds ratio; CI, Confidence interval.

A total of 5 publications reported the 1-year OS, 2-year OS and 3-year OS, which was not significantly different between the TA+LR group and LR group at 1-year (OR=0.88, 95% CI: 0.42–1.82; p=0.72; **Figure 2A**), 2-year (OR=0.72, 95% CI: 0.49–1.04; p=0.08) (**Figure 2B**), 3-year (OR=0.95, 95% CI: 0.48–1.88; p=0.88) (**Figure 2C**).

### Disease-free survival

**Figure 3:**
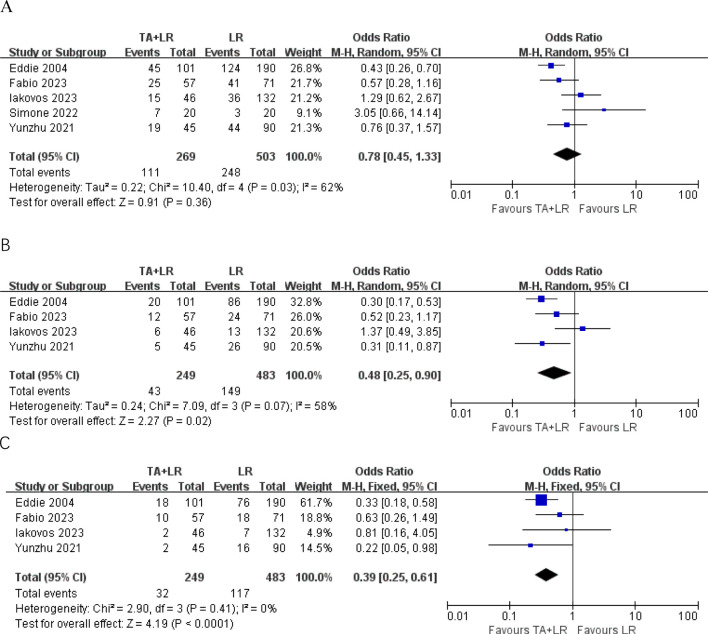
Forest plots comparing disease-free survival rates between TA+LR and LR groups. **(A)** Pooled analysis of the 1-year disease-free survival rate. **(B)** Pooled analysis of the 2-year disease-free survival rate. **(C)** Pooled analysis of the 3-year disease-free survival rate. OR, Odds ratio; CI, Confidence interval.

A total of 5 publications reported the 1-year DFS, disease-free survival (DFS) did not differ significantly between groups at 1-year (OR=0.78, 95% CI: 0.45–1.33; p=0.36, **Figure 3A**). Patients in the TA+LR group had significantly shorter 2-year DFS (OR: 0.48, 95% CI: 0.25–0.90, P = 0.02, **Figure 3B**) and 3-year DFS (OR: 0.39, 95% CI: 0.25–0.61, P < 0.0001, **Figure 3C**).

### Subgroup analyses

#### Geographic region

**Figure 4:**
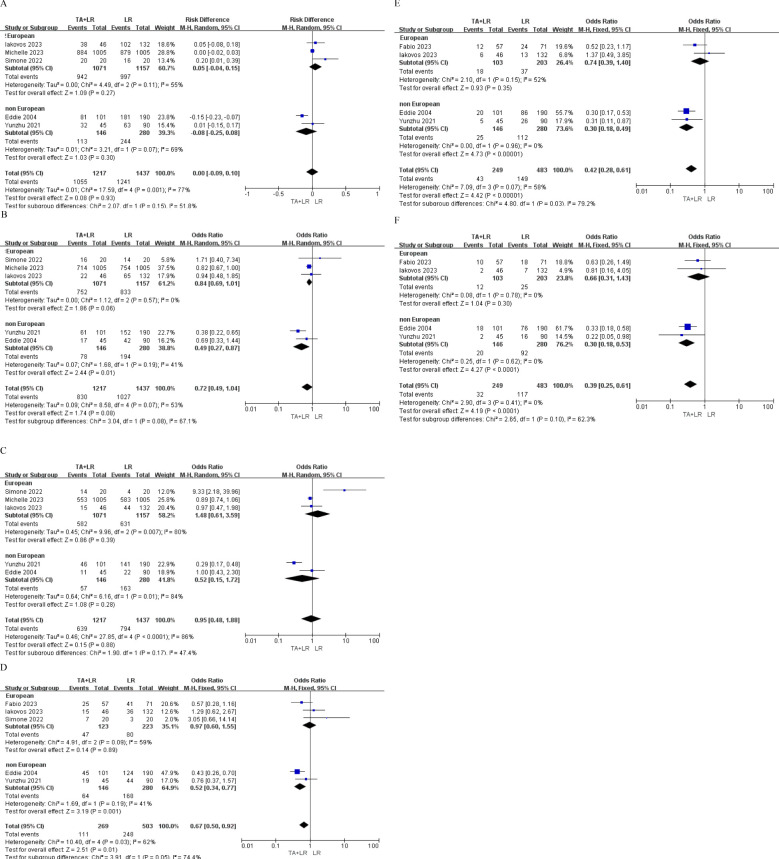
Subgroup analyses based on geographic region (European *vs*. Non-European studies). **(A)** Subgroup analysis comparing the 1-year overall survival rate. **(B)** Subgroup analysis comparing the 2-year overall survival rate. **(C)** Subgroup analysis comparing the 3-year overall survival rate. **(D)** Subgroup analysis comparing the 1-year disease-free survival rate. **(E)** Subgroup analysis comparing the 2-year disease-free survival rate. **(F)** Subgroup analysis comparing the 3-year disease-free survival rate. OR, Odds ratio; CI, Confidence interval.

Subgroup analyses based on geographic region (European *vs*. non-European studies) revealed no significant survival differences (all p>0.05) at 1-year, 2 -year and 3-year OS (**Figures 4A–C**). However, patients in the TA+LR group gained significantly shorter 1-year, 2 -year and 3-year DFS in non-European studies subgroup (**Figures 4D–F**).

### Ablation modality

**Figure 5:**
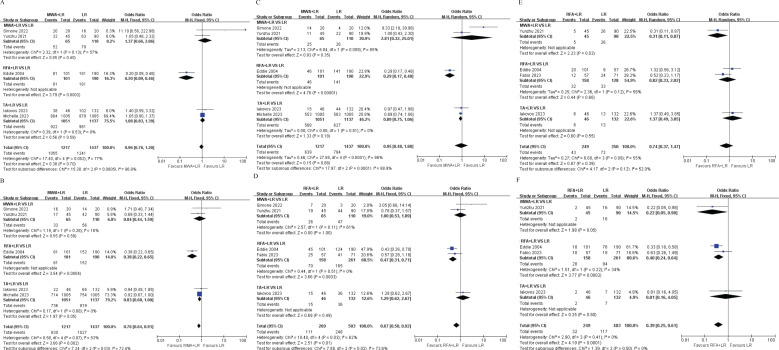
Subgroup analyses based on ablation modality (Microwave Ablation [MWA] *vs*. Radiofrequency Ablation [RFA] *vs*. Thermal Ablation [TA]). **(A)** Subgroup analysis of the 1-year overall survival rate. **(B)** Subgroup analysis of the 2-year overall survival rate. **(C)** Subgroup analysis of the 3-year overall survival rate. **(D)** Subgroup analysis of the 1-year disease-free survival rate. **(E)** Subgroup analysis of the 2-year disease-free survival rate. **(F)** Subgroup analysis of the 3-year disease-free survival rate. OR, Odds ratio; CI, Confidence interval.

In our study, we grouped RFA (Radiofrequency Ablation) and MWA (Microwave Ablation) together under the term TA (Thermal Ablation). In some of the included studies, the specific modality of thermal ablation was not explicitly specified. Therefore, we analyzed TA as a separate subgroup. Subgroup analyses based on ablation modality (MWA *vs* RFA *vs*. TA) revealed patients in the RFA+LR group gained significantly shorter 1-year, 2 -year, 3-year OS and 1-year, 3-year DFS than patients in LR group (**Figures 5A–F**).

### Safety

**Figure 6:**
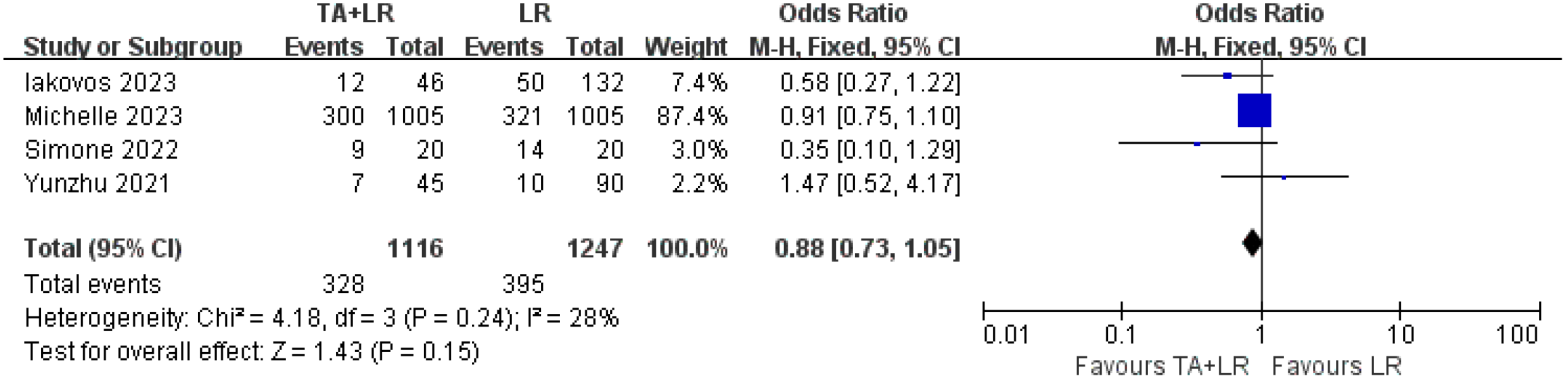
Comparative analysis of postoperative complications in TA+LR and LR groups. This figure presents a pooled analysis comparing the incidence of postoperative complications between patients in the TA+LR group and those in the LR group. A fixed effects model was applied for the analysis. Each horizontal line indicates the study-specific hazard ratio and its corresponding 95% confidence interval. The size of the squares represents the relative weight of each study in the analysis, while the diamond shape denotes the overall pooled odds ratio and its 95% confidence interval. OR, Odds ratio; CI, Confidence interval.

Four of the included studies compared the complication between the TA+LR group and LR group. There was no significant difference in the incidence of postoperative complications between the TA+LR group and LR group (OR: 0.88, 95% CI: 0.73–1.05, P = 0.15, **Figure 6**).

### Sensitivity analysis

**Figure 7:**
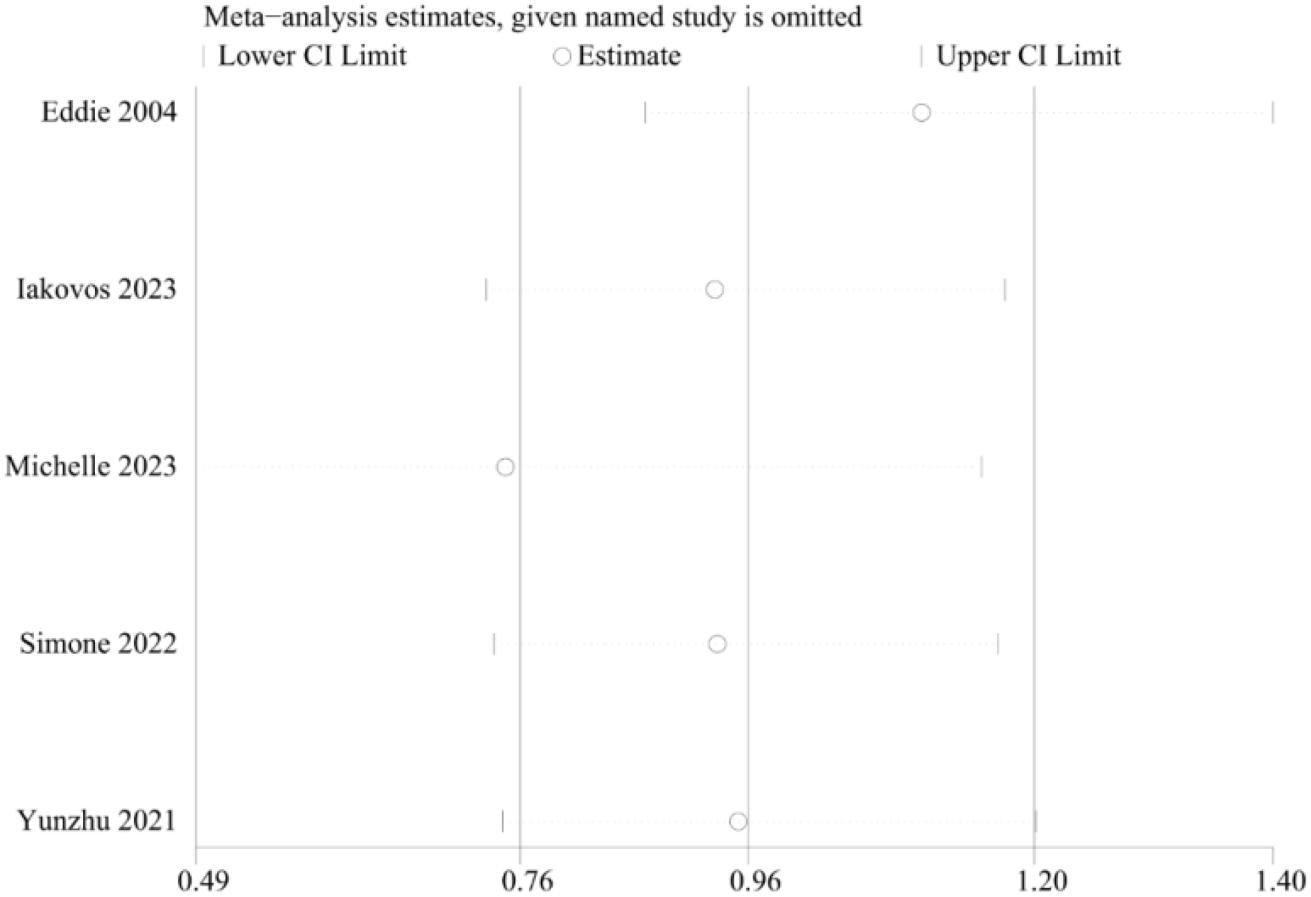
Sensitivity analyses of the survival rate comparisons between patients in the TA+LR and LR groups.

Sensitivity analysis was carried out via the leave-one-out approach. This analysis indicated that the exclusion of any single study did not significantly affect the pooled results on the overall survival results. The results of the meta-analysis were therefore concluded to be stable and reliable (**Figure 7**).

### Publication bias

**Figure 8:**
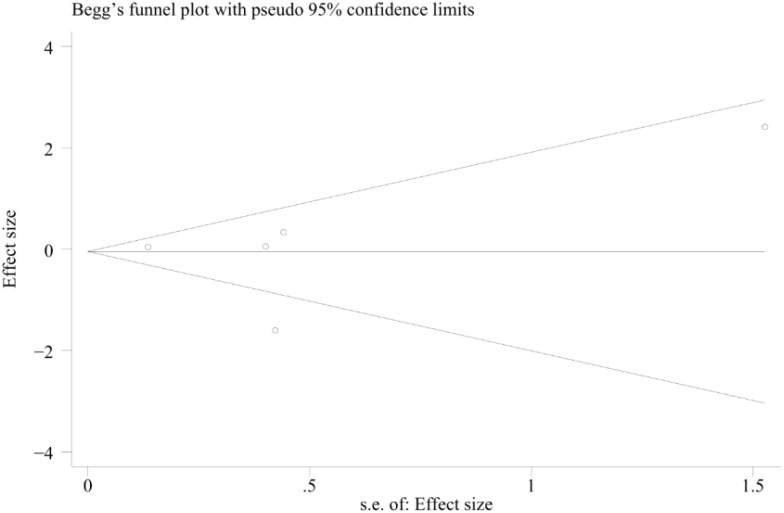
Funnel plot describing the comparative analysis of survival rates between patients in the TA+LR and LR groups.

We assessed the funnel plot of the total complications for any publication bias. The funnel plot was symmetrical, indicating a lack of publication bias (p= 0.851) (**Figure 8**).

## Discussion

Colorectal cancer (CRC) frequently progresses to liver metastases, a condition observed in approximately 50% of cases. Current therapeutic strategies for solitary colorectal liver metastases (CRLM) include surgical resection, radiofrequency ablation (RFA), cryosurgery, hepatic arterial infusion, and systemic chemotherapy. The integration of chemotherapy, molecular targeted therapy, and radiotherapy has extended the median survival time to 24 months; however, the five-year overall survival (OS) rate remains disappointingly low for patients not undergoing surgical intervention. Surgical resection remains the gold standard for CRLM, with complete tumor resection (R0) being the recommended strategy (17). However, liver resection is often precluded by factors such as large tumor volume, multiple metastases, complex anatomical locations, or poor patient health. Thermal ablation (TA), with its minimally invasive nature, may offer an advantage in locally controlling CRLM (18). Moreover, advancements in imaging-guided localization and probe technology have significantly expanded the indications for TA.

### Meta-analysis insights

This meta-analysis, encompassing 3,084 patients across six high-quality retrospective studies, provides critical insights into the evolving multimodal treatment paradigm for multifocal CRLM. The core finding is that combined thermal ablation and liver resection (TA+LR) is comparable to liver resection alone (LR) in terms of long-term survival rates, with no significant differences in overall survival (OS) at all time points (1–3 years), consistent with new evidence from propensity-matched analyses. Importantly, major complication rates were similar between the two groups (OR: 0.88, 95% CI: 0.73–1.05, P = 0.15), reinforcing TA+LR as a viable option for marginally resectable patients and challenging the traditional hierarchy that prioritizes resection as the sole curative approach. While combined resection and ablation is not superior to liver resection alone, it provides an effective treatment choice for patients who are not candidates for extensive liver resection, especially those with anatomically challenging lesions or limited future liver remnant (FLR). However, the decision to combine thermal ablation with surgical resection should be individualized based on patient-specific factors, including tumor burden, liver function, and overall functional status. Furthermore, liver-sparing hepatectomy allows for repeat resection in case of intrahepatic recurrence, thereby increasing the possibility of salvage surgery. For advanced CRLM patients with multiple metastases where R0 resection is deemed impossible, the combination of thermal ablation and liver resection has expanded the boundaries of CRLM resectability, preserving liver parenchyma while broadening the scope of curative surgery.

It is worth noting that, although there were no significant differences in overall survival (OS) at various time points (1–3 years), disease-free survival (DFS) was worse in the combined treatment group. Potential mechanisms may include the following: First, residual micrometastases in the ablation zone may accelerate intrahepatic recurrence without immediately affecting the patient’s overall survival, as salvage treatments for local recurrence are effective (19). Second, the technical limitations of TA in achieving histologically confirmed margins—especially for deep-seated perivascular lesions—may allow residual microscopic disease, a key factor since R1 resection margins independently predict DFS in CRLM. Most CRLM patients will develop recurrent disease after the first local treatment (20). However, there are discrepancies in the results regarding intrahepatic local recurrence, with patients treated with combined resection and ablation at a disadvantage. Yet, a recent retrospective study found a weak correlation between overall survival and recurrence-free survival, and the development of recurrent CRLM after liver resection does not necessarily rule out the possibility of cure. When feasible, repeat local treatment for recurrent disease has shown similar survival rates to those after the first liver resection.

### Subgroup analysis insights

Subgroup analysis revealed a steeper DFS decline in non-European TA+LR cohorts than in LR cohorts, possibly reflecting regional differences in the sequence of adjuvant therapy. For instance, Asian centers typically administer neoadjuvant chemotherapy before the ablation-resection sequence, while European guidelines prioritize upfront surgery for resectable disease (21). Subgroup analysis also showed that DFS was significantly lower in the radiofrequency ablation (RFA) subgroup compared to the microwave ablation (MWA) subgroup, likely due to the heat-sink effect of RFA on perivascular lesions and sublethal thermal injury-induced phenotypic transformation of tumor cells. Previous studies have shown that microwave ablation is less affected by tumor location and tissue type because of its lower sensitivity to the heat-sink effect. Moreover, microwave ablation can produce a more predictable, larger, and spherical ablation zone in a shorter time (22). Chong et al. (23)recently published their randomized McRFA trial, confirming that microwave ablation is as safe and effective as radiofrequency ablation for small hepatocellular carcinoma (HCC) lesions. Qiang Zhu and colleagues found significantly better DFS with microwave ablation compared to radiofrequency ablation in propensity score-matched patient groups with small perivascular HCC (24). A recent meta-analysis found a significantly lower local recurrence rate (ASR) in laparoscopic microwave ablation (OR: 2.16) compared to laparoscopic radiofrequency ablation, with no significant difference in major complication rates (OR: 0.21) (25).

### Clinical practice implications

From a clinical practice perspective, the subgroup analysis of this study provides three optimization pathways: First, multidisciplinary decision-making should prioritize ensuring FLR>30%, using three-dimensional CT reconstruction and indocyanine green clearance tests to select TA indications, providing an effective treatment choice for patients who are not candidates for extensive liver resection (26–28). Second, a standardized ablation technique protocol should be established, with MWA being the first choice for lesions >2cm or adjacent to large blood vessels (within 3mm of the main portal vein), and intraoperative contrast-enhanced ultrasound should be used to verify ablation margins (>5mm) to reduce the risk of local recurrence. Third, a dynamic monitoring plan should be implemented, recommending CT/MRI combined with CEA testing (sensitivity 92.4%) every three months for the first two years after TA+LR to identify intervenable intrahepatic recurrence early (29, 30). Notably, geographical differences show that the strategy of using neoadjuvant chemotherapy combined with staged ablation may weaken the DFS disadvantage (OR=0.97 *vs*. 0.52 in the European cohort). This suggests that the synergistic effect of treatment timing and regional diagnostic and treatment guidelines (such as the differences between ESMO and CSCO guidelines) needs to be considered in decision-making (31, 32).

### Limitations and future directions

This study has several limitations. Most included studies were retrospective, and long - term outcome studies on combined resection and ablation are rare. The retrospective design also led to insufficient control of unmeasured confounding factors such as RAS/BRAF mutation status, and the lack of histopathological data from the ablation zone made it difficult to objectively assess margin sufficiency. We initially noted the potential for bias and residual confounding. To elaborate, RAS/BRAF mutations can significantly influence CRLM prognosis and treatment response. Without data on these mutations, our results might be confounded, and our understanding of treatment effectiveness could be limited. Second, there was considerable heterogeneity in the modalities and protocols of TA. The variability in ablation parameters, such as power settings and treatment duration, was notable across the studies. For instance, microwave ablation (MWA) energy output ranged from ≥60W in some studies to lower levels in others, while radiofrequency ablation (RFA) protocols also differed in terms of applied power and treatment time. These differences in ablation parameters can significantly influence treatment efficacy and safety.

To address these limitations, we recommend future studies incorporate stratification based on molecular profiles such as RAS/BRAF mutations. This could provide more precise treatment effect estimates and help patient identify subgroups that may benefit most from specific therapies. Future research should also have larger sample sizes, prospective designs, and stricter control of confounding factors. RCTs and cohort studies are needed to confirm our findings and clarify the role of combined resection and ablation in CRLM treatment.

## Conclusion

The integration of thermal ablation (TA) with liver resection represents a viable liver-sparing approach for the management of extensive colorectal liver metastases (CRLM), applicable via both laparoscopic and open surgical techniques. This combined modality should be considered as a therapeutic alternative to liver resection alone, particularly for patients presenting with multiple metastases. However, the current body of research delineating the role and efficacy of TA within multimodal CRLM treatment regimens remains limited, especially when juxtaposed against modern chemotherapy protocols and advanced surgical methodologies. Consequently, there is a pressing need for prospective, multicenter randomized controlled trials (RCTs) or other high-caliber studies to more precisely delineate the therapeutic niche of TA in CRLM management.

## Data Availability

The original contributions presented in the study are included in the article/supplementary material. Further inquiries can be directed to the corresponding author.
